# Beauty and Social Interest Matter: Effects of Male’s Facial Attractiveness, Vocal Attractiveness and Social Interest on Female’s Decisions in Three-Person Games

**DOI:** 10.3390/bs14121154

**Published:** 2024-12-02

**Authors:** Junchen Shang, Yizhuo Zhang

**Affiliations:** Department of Medical Humanities, School of Humanities, Southeast University, Nanjing 211189, China; 220224090@seu.edu.cn

**Keywords:** facial attractiveness, vocal attractiveness, social interest, beauty premium, third-party punishment

## Abstract

Facial attractiveness, vocal attractiveness, and social interest influence two-person decision making. However, it remains unclear how these three factors jointly influence three-person bargaining. We investigated the impact of facial attractiveness, vocal attractiveness, and social interest on fairness decisions in a three-person ultimatum game and a third-party punishment dictator game. The results of the ultimatum game showed that in the condition of positive social interest, the acceptance rate of unfair/fair offers was higher when third players had attractive faces or attractive voices. Attractive faces of third players also increased acceptance rates of unfair/unfair offers than unattractive faces when third players expressed negative social interest. In the third-party punishment game, participants rated unfair allocations from unattractive-voice proposers with attractive faces (compared to unattractive faces) and proposers who expressed negative social interest (compared to positive social interest) as more reasonable. Regarding the punishment intentions, among the three factors, both the effect of vocal attractiveness and social interest were modulated by the combinations of the other two factors, while the effect of facial attractiveness remains robust across all conditions. These findings suggest that fairness decisions in three-person bargaining games are affected by beauty premium and social interest, while these effects vary across different decision contexts.

## 1. Introduction

As the saying goes, “The golden rule of conduct is mutual fairness”. Adhering to fairness is a vital quality that people universally exhibit in various social interactions [1]. Fairness also contributes to cooperation and success among groups [2]. As a general concept, fairness encompasses many notions. A social preference model assumes that individuals compare their own interests with those of others or with the average interest of groups. If they perceive themselves as wealthier or poorer, they will feel a sense of unfairness [3,4]. As in most economic decision-making research [5,6,7,8], fairness refers to equal distribution in this study. Furthermore, human beings show a strong aversion to inequal distribution of assets and are willing to impose punishment on individuals who engage in unfair behaviors [9,10].

Multiple previous studies have found that individuals’ fairness decisions could be influenced by facial attractiveness in economic exchanges, indicating the presence of a “beauty premium” [11]. For example, Farrelly et al. [12] confirmed that in both the two-person dictator game and two-person ultimatum game, participants were more cooperative towards opposite-sex players who had attractive faces. Similarly, Ma et al. [5,6] revealed that offers from attractive proposers were more likely to be accepted compared to those from unattractive proposers. Recent research revealed that attractive voices could induce a similar beauty premium effect to attractive faces in economic transactions. For instance, Shang and Liu [7,8] showed that participants made more investments in the partners with attractive voices than unattractive voices in two-person trust games. Therefore, the economic advantages stemming from facial or vocal attractiveness prevail in two-person games.

However, economic decision making is often more complex in real life, and involves more than two persons. Some researchers used three-person bargaining games to explore the beauty premium effect, for example, the three-person ultimatum game (TUG) [13], involving a proposer, a recipient, and a powerless third player. The proposer suggests how to split the given money; the recipient has veto power to decide whether to accept the allocation for both themself and the third player based on the monetary allocation from the proposer and relevant information from the third player. While the third player has no authority to allocate money like the proposer, nor can they reject or accept allocations like the recipient, they can only accept the final decision made by the recipient. Only the appearance of the proposer is important in the first-person dynamics in the ultimatum game, while both the appearances of the proposer and the third player are important for the third-person dynamics. Using this game, Ma and Hu [14] found the acceptance rate of unfair/fair offers was higher when the third player had an attractive face than an unattractive face, even the offers were unfair to the participant but fair to the third player. Attractive voices also increased participants’ acceptance rates of unfair/fair proposals in the TUG [15].

The beauty premium has also been found in the third-party punishment dictator game (TDG) [10], in which participants are no longer stakeholders as proposers or recipients, but instead, they act as interest-free third parties evaluating the fairness of asset distribution and the punishment intentions to proposers. Li and Zhou [10] showed that same-sex proposers were punished more severely than opposite-sex proposers when they had attractive faces, but opposite-sex proposers were punished more severely than same-sex proposers when they had unattractive faces, indicating that the facial attractiveness of proposers, along with their gender, jointly influence decision making. Using the same paradigm, Shang et al. [16] stated that attractive-voice proposers were rated as more reasonable in allocations and received lighter punishment for unfair allocations, compared to unattractive-voice proposers. In conclusion, these studies have demonstrated that both facial attractiveness and vocal attractiveness influence individuals’ fairness decisions in the context of three-person games.

While the beauty premium of faces and voices on decision making have been examined individually, as mentioned above, there is limited research exploring the integrated effects of the two factors from an audiovisual integration perspective. Previous research has identified a “face dominance” effect in cognitive processing using a Stroop-like paradigm, where facial attractiveness from the visual channel exerted a greater impact than voice attractiveness from the auditory channel [17]. Faced with face–voice pairs with congruent or incongruent attractiveness information, participants were instructed to judge the attractiveness of each channel separately, while ignoring the information from the other channel. The results demonstrated that judgments of vocal attractiveness were affected by unconscious facial attractiveness at both early perceptual encoding stages and later evaluative stages. However, judgments of facial attractiveness were only influenced by unconscious vocal attractiveness during the early perceptual encoding stage. Additionally, Rezlescu et al. [18] and Well et al. [19] both found that in the overall attractiveness ratings of face–voice pairs, the contribution of faces outweighed that of voices. Thus, these findings raise the question of whether “face dominance” would still be prominent in decision making if both facial and vocal attractiveness were effectively manipulated. In this study, we aimed to investigate this question.

Furthermore, the effect of attractiveness may be modulated by other factors such as social interest. Social interest signifies important social cues that are conveyed to others, indicating interest or disinterest [20,21]. Social interest can be represented through various means, including facial expressions, gaze direction, and semantic content [22]. Among these, manipulating social interest through semantics has been shown to be a relatively effective method: “I like you” expresses positive social interest, while “I don’t like you” indicates negative social interest [20]. For instance, using the two-person ultimatum game, Yuan et al. [22] found that acceptance rates of unfair offers were lower for negative social interest (than positive social interest) and lower for unattractive voices (than attractive voices). Moreover, Shang and Zhang [23] investigated the opposite-sex effects of social interest and integrated audiovisual attractiveness in the ultimatum game and dictator game, which both involved two players: the proposer and the recipient. The results showed that female proposers allocated more money to males who expressed positive social interest compared to those who expressed negative social interest. While as recipients, females expected males expressing positive interest to offer them more money compared to males expressing negative interest. This indicates that the effect of social interest may vary with participants’ roles. In addition, when males expressed positive social interest, female proposers allocated more money to attractive-voice males than unattractive-voice males, while this effect was absent when males expressed negative interest. These findings demonstrate that both social interest and the beauty premium influence fairness decisions in two-person games. But in three-person bargaining games, it remains unclear how these factors matter together, which was the second question that we aimed to examine.

Additionally, attractiveness not only influences human behavior in mate selection but also leads to gender differences in fairness decision making. For example, Li and Zhou [10] found that for unattractive-face proposers, female participants were more willing to punish opposite-sex proposers than same-sex counterparts, compared with male participants. Shang and Liu [24] also observed that in the two-person ultimatum game decisions involving vocal attractiveness, women were more influenced by the effect of vocal attractiveness than men. Specifically, this was reflected in females’ higher acceptance of offers from attractive-voice proposers, while men did not show the same behavior. However, the study adopted a four-factor within-subject design; it is too complex to include gender factors. Therefore, this research explored the effect of males’ attractiveness and social interest on females’ fairness decisions in three-person games. The reason for using only the combination of male stimuli and female participants is simply an issue of availability.

Hence, we utilized the same experimental materials (male faces and male voices) as in the previous study [23]. Social interest was manipulated through male voices with different semantics: “I like you” expressed positive social interest, while “I don’t like you” expressed negative social interest [20,22]. The present study employed two very different tasks: a three-person ultimatum game (TUG) and a third-party punishment dictator game (TDG). Specifically, this research examined the extent to which facial attractiveness, vocal attractiveness, and social interest play a role in two different cross-sex decision contexts which are both relevant to bargaining behaviors in a biological market. In the TUG, the participants receive information about the third players (their faces, voices, and social interest). The participants then make a decision about what monetary outcomes they, the proposer, and the third player, should receive. Therefore, the TUG focuses on the effects of how a person’s attractiveness and social interest affect how participants treat others. The TDG gives the participant attractiveness and social interest information about the proposer, and then the participant judges how the proposer treated the recipient. Hence, the TDG emphasizes the effects of a person’s attractiveness and social interest on how participants judge others. The motivation to use these two tasks is twofold. On the one hand, to expand existing research on attractiveness and social interest from two-person games [23] to three-person games. On the other hand, we sought to investigate how attractiveness and social interest work in concert in two different decision processes, i.e., how we treat and judge others, providing insights into the practical factors that impact fair decision making. However, it should be noted that this study is also an exploratory attempt for hypothesis generation. Additionally, to simplify the experimental design, we only manipulated the appearance of one of the people in the game.

The research hypotheses of the TUG are as follows:

**H1.**  *Attractive faces and voices would increase participants’ acceptance rates, replicating the beauty premium observed in prior studies [6,15]*.

**H2.**  *The acceptance rate would be higher when players expressed positive social interest compared to those who exhibited negative social interest [22]*.

The research hypotheses of the TDG are as follows:

**H3.**  *Unfair allocations from proposers with attractive faces or attractive voices or expressing positive social interest would be rated as more reasonable [10,16,22]*.

**H4.**  *Attractive proposers or proposers expressing positive social interest may be punished less harshly when they make unfair allocations [10,16,22]*.

In addition, there are two exploratory hypotheses regarding two tasks:

**H5.**  *According to the “face dominance” phenomenon [17], we anticipated a difference between the effect of facial attractiveness and impact of vocal attractiveness on decision making.*

**H6.**  *We expected an interaction among facial attractiveness, vocal attractiveness, social interest, and fairness. Especially, the effect of attractiveness may be modulated by social interest, based on the findings in two-person bargaining games [23]*.

## 2. Methods

### 2.1. Participants

Using parameters from prior research [23], a sample size of 52 participants was calculated using the More*Power 6.0.4 software [25], with a statistical test power of 0.80 at *α* = 0.05 and a large effect size (*η_p_*^2^) of 0.14. Moreover, based on previous studies on the effect of facial or vocal attractiveness on social decision making [22,26], we finally recruited 70 female students (*M_age_* = 21.23 years, ranging from 18 to 26, *SD* = 2.00) from Southeast University who were not majoring in economics or psychology. Data from two participants were excluded since they did not seem to consider any information, such that they chose the acceptance option throughout all trials of the TUG. All participants were right-handed and reported normal or corrected-to-normal vision and normal hearing, without physical or mental illness. They were informed that they would be remunerated including a basic payment plus the payoff from two randomly selected trials during the three-person ultimatum game. Actually, all participants received the same amount of compensation after the experiment regardless of their performance [22,27]. All subjects signed informed consent before the experiment.

The current research was approved by the Ethics Committee of the Psychology Research Center at Southeast University (NO: 20231230) and all research was performed in accordance with the Declaration of Helsinki and relevant guidelines.

### 2.2. Design and Materials

The present study adopted a four-factor within-subject design, with facial attractiveness (attractive vs. unattractive), vocal attractiveness (attractive vs. unattractive), social interest (positive vs. negative), and fairness (TUG: fair/fair, fair/unfair, unfair/fair, unfair/unfair; TDG: fair vs. unfair) as within-participant variables. The dependent variable in the TUG was the participants’ acceptance rates of offers, while in TDG the ratings of the allocations’ reasonableness and the punishment intentions to proposers were the dependent variables. In the TUG, fairness was defined by how the proposer allocated CNY 12 between the proposer and recipients (i.e., the participant and the third player): fair/fair offer (each person received CNY 4), fair/unfair offer (the participant received CNY 4, and the third player received CNY 1; the proposer received CNY 7), unfair/fair offer (the participant received CNY 1, and the third player received CNY 4; the proposer received CNY 7), and unfair/unfair offer (the participant and the third player both received CNY 1, the proposer received CNY 10). In the TDG, fairness was defined by how much out of CNY 10 the proposer allocated to the recipient. Allocations of CNY 5 or CNY 4 were considered fair, CNY 1 or CNY 2 were considered unfair. CNY 3 was considered mildly unfair, such allocations were used as fillers [10].

The face stimuli were selected from Shang and Zhang’s research [23], with 32 neutral faces (16 attractive male faces and 16 unattractive male faces). These pictures were converted to grayscale, removed extra features such as clothing and necks, and resized to a unitary size (350 × 450 pixels, 4.3 × 6.1 cm, 4.2° × 5.5° visual angles) in Adobe Photoshop 2022. All subjects reported being unfamiliar with the people in the photos (no celebrities). According to Shang and Zhang [23], nine-point Likert rankings were made on the attractiveness of all faces (1 = “very unattractive”, 9 = “very attractive”). One-way ANOVA for the ratings revealed a significant difference in attractiveness, *F*(1,30) = 199.90, *p* < 0.001, *η_p_*^2^ = 0.87, with attractive faces being perceived as more attractive (*M* = 6.37, *SD* = 0.44) than unattractive ones (*M* = 3.38, *SD* = 0.33).

The voice stimuli were different for positive and negative social interest. They were collected from prior research [23], with 32 voices in neutral mood (16 attractive male voices and 16 unattractive male voices). Among each level, 8 had positive social interest (which read “我喜欢你”, Chinese Pinyin: “wo xi huan ni, meaning “I like you”) and 8 had negative social interest (which read “我不喜欢你”, Chinese Pinyin: “wo bu xi huan ni, meaning “I don’t like you”). It is noteworthy that the social judgment was towards nobody in particular, which is a limitation of the design. The durations of all voice materials ranged from 800 to 1200 ms. All sounds were recorded at the intensity of 70 dB, without any poor articulations or noises. Also, the attractiveness of these voices was rated on a nine-point scale (1 = “very unattractive”, 9 = “very attractive”) [23]. A 2 (vocal attractiveness: attractive, unattractive) × 2 (social interest: positive, negative) ANOVA was conducted on the mean ratings of attractiveness. Only the main effect of vocal attractiveness was significant, *F*(1,28) = 225.85, *p* < 0.001, *η_p_*^2^  = 0.89, indicating that attractive voices were rated as more attractive than unattractive ones. In addition, to exclude the confounding effect with the participants’ emotional valence, the pleasure and arousal of voices were also rated on two 9-point scales (1 = “the least pleasant/the least awakened or excited”, 9 = “the most pleasant/the most awakened or excited”) [23]. In ratings of pleasure and arousal, the main effect of attractiveness and social interest, as well as their interaction, were not significant, *F*s ≤ 4.05, *p*s ≥ 0.054. The descriptive statistics for four voice groups about ratings of vocal attractiveness, pleasure, and arousal are shown in Table 1.

Since the participants who provided their photographs and voices would not like their information to be available online, the materials of this article will be made available by the corresponding author on reasonable request.

### 2.3. Procedure

Participants comfortably sat in a sound-attenuated laboratory, with a viewing distance of 55 cm, wearing Sennheiser HD201 headphones, to complete the experiment. Before the experiment, the listening volume was adjusted for each participant for comfort. The stimulus presentation and measurements were controlled by E-prime 3.0 on a 14-inch-screen Lenovo ThinkPad laptop (60 Hz refresh rate, 2440 × 1400 pixels resolution).

There were two experimental tasks: a three-person ultimatum game (TUG) [15] and a third-party punishment dictator game (TDG) [10,16]. The order of the TUG and TDG was counterbalanced between participants. We informed participants that all players in the two games were local university students whose decisions had been collected in another experiment. Their decisions in each trial of the game would not affect the next trial. There were 8 practice trials in the TUG and 10 practice trials in the TDG before the formal experiment. The faces and voices in the practices would not appear in formal experiments.

**Figure 1:**
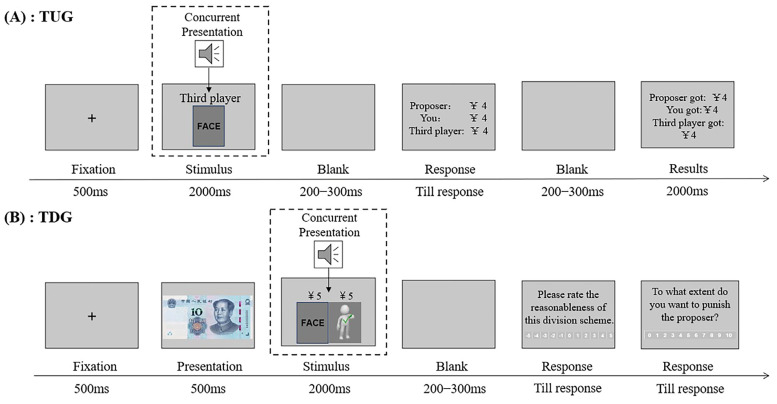
Schematic representation of three-person ultimatum game (TUG, see (**A**)) and third-party punishment dictator game (TDG, see (**B**)).

The experimental procedure of the TUG is illustrated in Figure 1A. Firstly, a fixation appeared for 500 ms. Then, a face–voice pair of the third player was presented for 2000 ms, followed by a blank screen for 200–300 ms. Subsequently, the proposer’s offer of how to split CNY 12 among the three persons was displayed. The participants acting as recipients were given unlimited time to decide whether to accept allocations for themselves and another third player by pressing two keys on the keyboard (“A” for accepting and “L” for rejecting; the key order was reversed for half of the participants). If the allocation was accepted, all three people would receive the money as proposed; otherwise, none of them would receive anything. The third players had no choice regarding allocations. Once a decision was made, after a blank screen for 200–300 ms, the final allocation outcome of that trial was displayed on the screen for 2000 ms. Then, the next trial started. A total of 32 face–voice pairs (with eight conditions) and 4 fairness levels were combined. Each combination of face–voice pair and allocation was presented once in a random order in one block and was repeated in another block. This amounted to a total of 64 experimental trials.

In the TDG, the participant acted as an interest-free third party to evaluate the reasonableness of the proposer’s allocations and express their intentions to punish the proposer. Participants were told that only proposers had the authority to make allocations, while recipients had to accept allocations. The experimental procedure is shown in Figure 1B. A fixation firstly appeared for 500 ms. Then, participants viewed a photo of a CNY 10 note in the center of the screen for 500 ms. After that, a frame which contained two people (a proposer with a face and a voice, and a recipient represented by a cartoon figure) and the allocation distributed by the proposer was presented for 2000 ms. After a blank screen lasting for 200–300 ms, participants were asked to complete two no-time-limit evaluation tasks sequentially on an 11-point scale using the mouse: (a) “please rate the reasonableness of this division scheme” (−5 = “very unreasonable”, 5 = “very reasonable”); (b) “to what extent do you want to punish the proposer” (0 = “not at all”, 10 = “very much”). Once the participant made the two decisions, the next trial started. A total of 32 face–voice pairs (with eight conditions) and 4 types of allocations (2 were fair, 2 were unfair) were combined. In addition, 8 moderately-attractive face–voice pairs (4 with positive and 4 with negative social interest) combined with a mildly unfair allocation (7/3) were used as fillers. These filler trials were discarded from the analysis [10]. Each combination of face–voice pair and allocation was presented once in a random order in one block and was repeated in another block. This amounted to a total of 80 experimental trials.

After the two games, participants rated the attractiveness of the 32 faces and 32 voices in the formal experiments separately using a 9-point Likert scale (1 = “very unattractive”, 9 = “very attractive”). The order of the face rating experiment and voice rating experiment was counterbalanced among participants.

At the beginning of each TUG trial, a fixation appeared for 500 ms. Next, the concurrent presentation of a face–voice pair was displayed for 2000 ms, followed by a blank screen for 200–300 ms. Then, viewing the allocation distributed by the proposer, the participant acted as the recipient and decided whether to accept the allocation for herself and another powerless third player. Once her decision had been made, after a blank screen for 200–300 ms, a 2000 ms final allocation outcome was presented. Then, the next trial started.

In the TDG, a fixation appeared at the beginning of each trial for 500 ms. Then, after a 500 ms presentation of a 10 yuan note, the participant viewed a frame lasting for 2000 ms which contained the proposer, the recipient, and an allocation distributed by the proposer. Next, a blank screen was displayed for 200–300 ms, followed by two no-time-limit evaluation tasks sequentially. The participant, acting as an impartial third party, evaluated the reasonableness of the proposer’s allocation and expressed her intention to punish the proposer. Once the participant made her two decisions, the next trial started.

### 2.4. Data Analysis

In TUG, a 2 (facial attractiveness: attractive, unattractive) × 2 (vocal attractiveness: attractive, unattractive) × 2 (social interest: positive, negative) × 4 (fairness: fair/fair, fair/unfair, unfair/fair, unfair/unfair) repeated measures ANOVA was used to compare participants’ acceptance rates of offers under different face and voice conditions.

In TDG, a 2 (facial attractiveness: attractive, unattractive) × 2 (vocal attractiveness: attractive, unattractive) × 2 (social interest: positive, negative) × 2 (fairness: fair, unfair) repeated measures ANOVA was conducted to compare participants’ ratings of allocations’ reasonableness and punishment intentions to proposers across varied conditions.

All data analysis was performed using IBM SPSS Statistics 26. The Greenhouse–Geisser method was conducted when the assumption of sphericity was not met. Our data are available on the GitLab (“https://jihulab.com/attractiveness/beauty-and-social-interest-matter (accessed on 1 December 2014)”).

## 3. Results

### 3.1. Evaluation Ratings of Facial and Vocal Attractiveness

We conducted a one-way ANOVA on the mean ratings of facial attractiveness. The ratings of attractive faces (*M* = 5.67, *SD* = 0.53) were significantly higher than unattractive faces (*M* = 2.98, *SD* = 0.47) (*F*(1,30) = 231.84, *p* < 0.001, *η_p_*^2^ = 0.89). Then, a 2 (vocal attractiveness: attractive, unattractive) × 2 (social interest: positive, negative) ANOVA was performed on the mean ratings of vocal attractiveness. The results found a significant main effect of vocal attractiveness (*F*(1,28) = 215.55, *p* < 0.001, *η_p_*^2^ = 0.89). Participants rated attractive voices (*M* = 6.27, *SD* = 0.12) as more attractive in comparison to unattractive voices (*M* = 3.78, *SD* = 0.12). Neither the main effect of social interest (*F*(1,28) = 0.24, *p* = 0.629, *η_p_*^2^ = 0.01), or the interaction between vocal attractiveness and social interest (*F*(1,28) = 0.04, *p* = 0.839, *η_p_*^2^ = 0.01) was significant. The post-experiment results demonstrate that the selected stimuli of faces and voices were effective.

### 3.2. Descriptive Statistics Results

The descriptive statistics for each condition’s outcome in the three-person ultimatum game (TUG) are presented in Table 2, and those for the third-party punishment dictator game (TDG) are shown in Table 3. In addition, the agreements of the outcomes across repetitions between two blocks for all experimental conditions in the two games were high (all Cronbach’s *α* ≥ 0.71).

### 3.3. Three-Person Ultimatum Game (TUG)

The ANOVA results for all effects in the TUG are shown in Table 4. The main effect of facial attractiveness was significant (*F*(1,67) = 17.15, *p* < 0.001, *η_p_*^2^ = 0.20), with a higher acceptance rate when third players had attractive faces (*M* = 0.64, *SD* = 0.02) than unattractive faces (*M* = 0.61, *SD* = 0.02). The main effect of vocal attractiveness was also significant (*F*(1,67) = 12.08, *p* = 0.001, *η_p_*^2^ = 0.15), indicating a higher acceptance rate when third players had attractive voices (*M* = 0.63, *SD* = 0.02) than unattractive voices (*M* = 0.61, *SD* = 0.02). The main effect of social interest was significant (*F*(1,67) = 17.11, *p* < 0.001, *η_p_*^2^ = 0.20), with a higher acceptance rate for third players expressing positive social interest (*M* = 0.66, *SD* = 0.02) compared to negative social interest (*M* = 0.59, *SD* = 0.02). In addition, the main effect of fairness was significant (*F*(2,154) = 254.89, *p* < 0.001, *η_p_*^2^ = 0.79). Post hoc comparisons further showed that acceptance rates for these offers differed significantly from each other (*p* ≤ 0.001), except that the difference between fair/fair offers and fair/unfair offers was not significant (*p* = 0.689). The acceptance rate was the highest for fair/fair offers (*M* = 0.98, *SD* = 0.01) and fair/unfair offers (*M* = 0.95, *SD* = 0.02). The second was unfair/fair offers (*M* = 0.36, *SD* = 0.03). The acceptance rate for unfair/unfair offers (*M* = 0.19, *SD* = 0.04) was the lowest. In addition, the interactions between facial attractiveness and social interest, vocal attractiveness and social interest, facial attractiveness and fairness, vocal attractiveness and fairness, and social interest and fairness were all significant (*F*s ≥ 6.61, *p*s ≤ 0.012).

**Figure 2:**
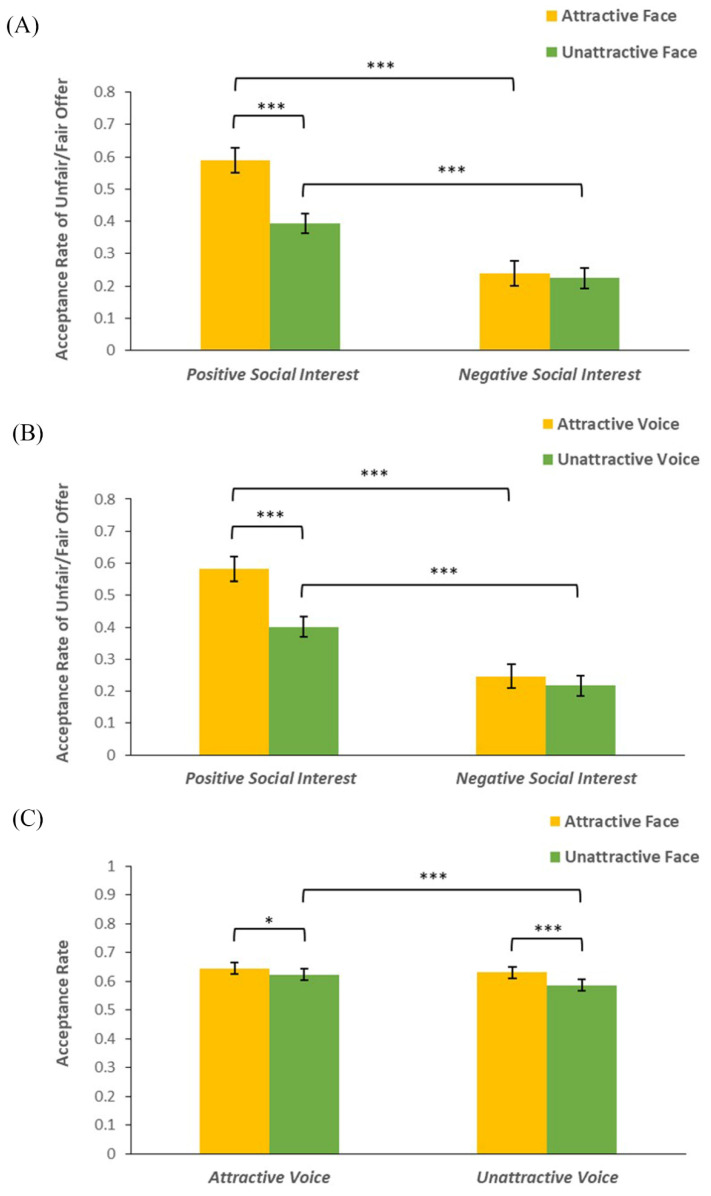
(**A**) The mean acceptance rates of unfair/fair offers as a function of facial attractiveness, social interest, and fairness. (**B**) The mean acceptance rates of unfair/fair offers as a function of vocal attractiveness, social interest, and fairness. (**C**) The mean acceptance rates as a function of facial attractiveness and vocal attractiveness. The error bars represent standard errors. * *p* < 0.05, *** *p* < 0.001.

Interestingly, the three-way interaction between facial attractiveness, social interest, and fairness was significant (*F*(2,151) = 11.45, *p* < 0.001, *η_p_*^2^ = 0.15). A further simple effect analysis revealed that under the condition of positive social interest, only the acceptance rate of unfair/fair offers was higher when third players had attractive faces than unattractive faces (see Figure 2A) (*F*(1,67) = 28.33, *p* < 0.001, *η_p_*^2^ = 0.30). Under the condition of negative social interest, only the acceptance rate of unfair/unfair offers was higher when third players had attractive faces than unattractive faces (see Figure 2A) (*F*(1,67) = 5.09, *p* = 0.027, *η_p_*^2^ = 0.07). Regardless of the level of facial attractiveness, only unfair/fair offers were accepted at significantly higher rates when third players expressed positive social interest than negative social interest (see Figure 2A) (*F*s ≥ 37.32, *p*s < 0.001). As for fairness, acceptance rates for offers differed significantly regardless of the combinations of facial attractiveness and social interest conditions (*F*s ≥ 119.28, *p*s < 0.001).

The three-way interaction between vocal attractiveness, social interest, and fairness was also significant (*F*(2,134) = 5.91, *p* = 0.003, *η_p_*^2^ = 0.08). A simple effect analysis found that under the condition of positive social interest, only the acceptance rate of unfair/fair offers was higher when third players had attractive voices than unattractive voices (see Figure 2B) (*F*(1,67) = 25.80, *p* < 0.001, *η_p_*^2^ = 0.28). Only unfair/fair offers were accepted at significantly higher rates when third players expressed positive social interest rather than negative social interest (see Figure 2B) (*F*s ≥ 36.55, *p*s < 0.001), regardless of the level of vocal attractiveness. As for fairness, acceptance rates for offers differed significantly regardless of the combinations of vocal attractiveness and social interest conditions (*F*s ≥ 123.32, *p*s < 0.001).

Moreover, there was a significant interaction between facial attractiveness and vocal attractiveness (see Figure 2C) (*F*(1,67) = 5.59, *p* = 0.021, *η_p_*^2^ = 0.08). A simple effect analysis revealed higher acceptance rates when third players had attractive faces comparing to unattractive faces, regardless of their vocal attractiveness (*F*s ≥ 6.64, *p*s ≤ 0.012). However, only under the unattractive-face condition did third players with attractive voices increase acceptance rates (*F*(1,67) = 14.27, *p* < 0.001, *η_p_*^2^ = 0.18). The difference in acceptance rates between attractive and unattractive voices was not significant under the attractive-face condition (*F*(1,67) = 3.70, *p* = 0.059, *η_p_*^2^ = 0.05).

### 3.4. Third-Party Punishment Dictator Game (TDG)

#### 3.4.1. Reasonableness Rating

The ANOVA results for all effects on reasonableness ratings in the TDG are shown in Table 5. The results show that participants’ reasonableness ratings were higher when proposers had attractive faces (*M* = −0.12, *SD* = 0.13) rather than unattractive faces (*M* = −0.22, *SD* = 0.13), (*F*(1,67) = 10.01, *p* = 0.002, *η_p_*^2^ = 0.13); when proposers expressed negative social interest (*M* = 0.19, *SD* = 0.18) rather than positive social interest (*M* = −0.53, *SD* = 0.16) (*F*(1,67) = 10.36, *p* = 0.002, *η_p_*^2^ = 0.13); and when allocations were fair (*M* = 2.16, *SD* = 0.24) rather than unfair (*M* = −2.50, *SD* = 0.19) (*F*(1,67) = 187.57, *p* < 0.001, *η_p_*^2^ = 0.74). However, the main effect of vocal attractiveness was not significant (*F*(1,67) = 0.91, *p* = 0.345, *η_p_*^2^ = 0.01). All interactions were non-significant (*F*s ≤ 1.45, *p*s ≥ 0.233) except for the following two interactions.

**Figure 3:**
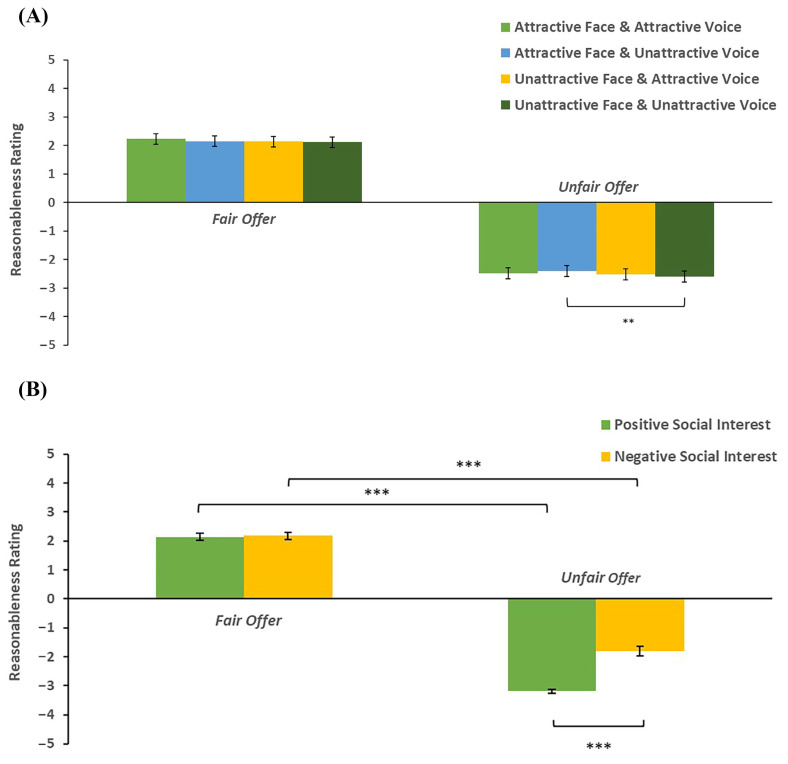
(**A**) The mean reasonableness ratings as a function of facial attractiveness, vocal attractiveness, and fairness. (**B**) The mean reasonableness ratings as a function of social interest and fairness. The error bars represent standard errors. ** *p* < 0.01, *** *p* < 0.001.

The three-way interaction between facial attractiveness, vocal attractiveness, and fairness was significant (*F*(1,67) = 3.96, *p* = 0.05, *η_p_*^2^ = 0.06). A simple effect analysis indicated that under the unattractive-voice condition, the reasonableness ratings of unfair offers were higher when proposers had attractive faces than unattractive faces (see Figure 3A) (*F*(1,67) = 11.67, *p* = 0.001, *η_p_*^2^ = 0.15). Under other conditions, simple effects of facial attractiveness on reasonableness ratings were not significant (*F*s ≤ 2.40, *p*s ≥ 0.126). Participants’ reasonableness ratings were higher for fair allocations comparing to unfair allocations, regardless of the attractiveness level of faces and voices (*F*s ≥ 165.69, *p*s < 0.001). However, none of the simple effects of vocal attractiveness on reasonableness ratings were significant across all conditions (*F*s ≤ 2.41, *p*s ≥ 0.126).

In addition, the two-way interaction between social interest and fairness was significant (see Figure 3B) (*F*(1,67) = 17.20, *p* < 0.001, *η_p_*^2^ = 0.20). A simple effect analysis revealed that unfair allocations were rated as less reasonable when proposers expressed positive social interest comparing to negative social interest (*F*(1,67) = 16.80, *p* < 0.001, *η_p_*^2^ = 0.20). Differences between fair offers and unfair offers were significant, no matter whether proposers expressed positive social interest (*F*s ≥ 72.58, *p*s < 0.001).

#### 3.4.2. Punishment Intention Rating

The ANOVA results for all effects on the punishment intention ratings in the TDG are shown in Table 6. The punishment intention rating of participants was lower when proposers had attractive faces (*M* = 3.04, *SD* = 0.21) rather than unattractive faces (*M* = 3.47, *SD* = 0.21), (*F*(1,67) = 39.03, *p* < 0.001, *η_p_*^2^ = 0.37); when proposers had attractive voices (*M* = 3.18, *SD* = 0.20) rather than unattractive voices (*M* = 3.33, *SD* = 0.21), (*F*(1,67) = 16.25, *p* < 0.001, *η_p_*^2^ = 0.20); and when allocations were fair (*M* = 1.59, *SD* = 0.17) rather than unfair (*M* = 4.92, *SD* = 0.29) (*F*(1,67) = 188.74, *p* < 0.001, *η_p_*^2^ = 0.74). The interactions between facial attractiveness and fairness and social interest and fairness were both significant, *F*s ≥ 13.19, *p*s ≤ 0.001. However, the main effect of social interest was not significant, (*F*(1,67) = 1.21, *p* = 0.276, *η_p_*^2^ = 0.02), and neither were any other two-way or three-way interactions (*F*s ≤ 2.90, *p*s ≥ 0.093).

In addition, a significant four-way interaction effect was observed in the ratings of participants’ punishment intentions (*F*(1,67) = 13.42, *p* < 0.001, *η_p_*^2^ = 0.17). As we planned to examine how facial attractiveness, vocal attractiveness, and social interest influenced participants’ punishment intentions, we separated the data into two sets (fair condition and unfair condition) in the further analysis.

**Figure 4:**
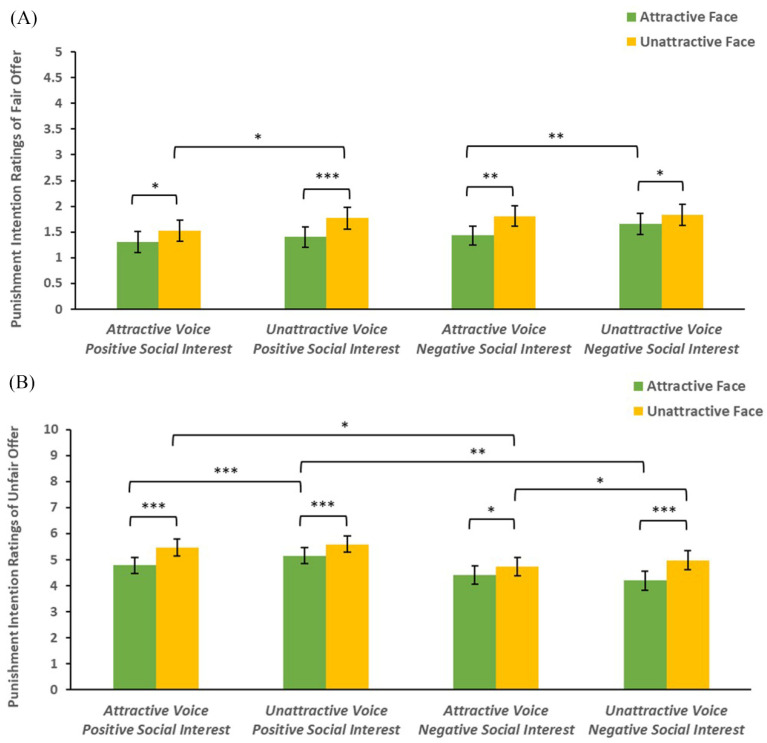
(**A**) The mean punishment intention ratings of fair offers as a function of facial attractiveness, vocal attractiveness, and social interest. (**B**) The mean punishment intention ratings of unfair offers as a function of facial attractiveness, vocal attractiveness, and social interest. The error bars represent standard errors. * *p* < 0.05, ** *p* < 0.01, *** *p* < 0.001.

In fair conditions, the main effects of facial attractiveness (*F*(1,67) = 20.56, *p* < 0.001, *η_p_*^2^ = 0.24) and vocal attractiveness (*F*(1,67) = 16.33, *p* < 0.001, *η_p_*^2^ = 0.20) were both significant. Participants were more willing to punish unattractive-face proposers (*M* = 1.73, *SD* = 0.18) than attractive-face proposers (*M* = 1.45, *SD* = 0.17), and more willing to punish unattractive-voice (*M* = 1.67, *SD* = 0.18) than attractive-voice proposers (*M* = 1.52, *SD* = 0.17). The three-way interaction between facial attractiveness, vocal attractiveness, and social interest was also significant (see Figure 4A) (*F*(1,67) = 4.26, *p* = 0.043, *η_p_*^2^ = 0.06). A simple effect analysis demonstrated that participants indicated a lower intention to punish attractive-voice proposers in comparison to unattractive-voice proposers when these proposers had attractive faces and expressed negative social interest (*F*(1,67) = 7.61, *p* = 0.007, *η_p_*^2^ = 0.10). Also, when proposers had unattractive faces and expressed positive social interest, participants showed lower intentions to punish attractive-voice proposers compared to unattractive-voice proposers (*F*(1,67) = 6.46, *p* = 0.013, *η_p_*^2^ = 0.08). The simple effects of facial attractiveness on punishment intention ratings were significant across all conditions (*F*s ≥ 4.00, *p*s ≤ 0.05), indicating that participants were less willing to punish attractive-face proposers than unattractive-face proposers.

In unfair conditions, the main effect of facial attractiveness was significant (*F*(1,67) = 38.55, *p* < 0.001, *η_p_*^2^ = 0.37): participants were more willing to punish unattractive-face proposers (*M* = 5.20, *SD* = 0.30) than attractive-face proposers (*M* = 4.64, *SD* = 0.29). The main effect of vocal attractiveness was significant (*F*(1,67) = 5.68, *p* = 0.02, *η_p_*^2^ = 0.08): participants were more willing to punish unattractive-voice (*M* = 4.98, *SD* = 0.30) than attractive-voice proposers (*M* = 4.85, *SD* = 0.29). The main effect of social interest was significant (*F*(1,67) = 4.71, *p* = 0.034, *η_p_*^2^ = 0.07): participants were more willing to punish proposers expressing positive social interest (*M* = 5.25, *SD* = 0.31) than negative social interest (*M* = 4.58, *SD* = 0.35). Also, the three-way interaction between facial attractiveness, vocal attractiveness, and social interest was significant (see Figure 4B) (*F*(1,67) = 10.02, *p* = 0.002, *η_p_*^2^ = 0.13). Further simple effect analysis revealed that participants showed a lower intention to punish attractive-voice proposers in comparison to unattractive-voice proposers when the proposers had attractive faces and expressed positive social interest (*F*(1,67) = 13.40, *p* < 0.001, *η_p_*^2^ = 0.17). When proposers had unattractive faces and expressed negative social interest, participants showed lower intentions to punish attractive-voice proposers compared to unattractive-voice proposers (*F*(1,67) = 4.75, *p* = 0.033, *η_p_*^2^ = 0.06). Interestingly, participants were more willing to punish proposers expressing positive social interest than negative social interest when proposers’ levels of facial attractiveness and vocal attractiveness mismatched (*F*s ≥ 5.28, *p*s ≤ 0.025). The simple effects of facial attractiveness on punishment intention ratings were significant across all conditions (*F*s ≥ 6.78, *p*s ≤ 0.011), indicating that participants were less willing to punish attractive-face proposers than unattractive-face proposers.

## 4. Discussion

This study examined how facial attractiveness, vocal attractiveness, and social interest influence women’s fairness decisions in a three-person ultimatum game and a third-party punishment game, and whether these effects vary with participants’ roles. By presenting third players’ faces and voices of different attractiveness levels to participants in the TUG, we found that under the condition of positive social interest, the acceptance rate of unfair/fair offers was higher when third players had attractive faces (compared to unattractive faces) or attractive voices (compared to unattractive voices). Furthermore, when third players expressed negative social interest and the offers were unfair for both participants and third players, attractive faces also increased acceptance rates, while the effect of vocal attractiveness did not exist. This suggests that the reward effect of attractive faces [28,29,30] is strong enough to attenuate participants’ feelings to inequality and their perception of negative social signals, while the effect of vocal attractiveness is obviously weaker. This provided new evidence for the effect of the beauty premium in three-person bargaining [14,15], although its impact was moderated by social interest cues.

In the TDG, the extent to which the beauty premium played a role was different for the reasonableness and punishment intentions. For reasonableness ratings, we found a weak influence of facial attractiveness, whereas no effect of vocal attractiveness existed. In the unattractive-voice condition, participants rated unfair allocations from proposers who had attractive faces (compared to unattractive faces) as more reasonable. This supports the previous research that participants were more objective in their perception of fairness when they knew objective information about the outcome of the behavior [10]. Nonetheless, for the ratings of punishment intentions, the impact of facial attractiveness was stable; that is, participants always showed lower willingness to punish proposers with attractive faces than unattractive faces no matter whether proposers had attractive voices or expressed positive social interest. Our findings are in line with previous research [10,26], demonstrating the robust “beauty premium” on faces. The impact of vocal attractiveness and social interest, similar to facial attractiveness, also diminished participants’ feelings of unfairness. However, this occurred under specific conditions and varied across different decision contexts. In fair conditions, participants indicated a lower intention to punish attractive-voice proposers (compared to unattractive-voice proposers) when they had attractive faces and expressed negative social interest, or when proposers had unattractive faces and expressed positive social interest. While in unfair conditions, participants showed a lower intention to punish attractive-voice proposers (compared to unattractive-voice proposers) when they had attractive faces and expressed positive social interest, or when proposers had unattractive faces and expressed negative social interest.

It is noteworthy that even if the beauty premium was observed in the three-person economic games, the effects were different between visual and auditory modalities. Especially, in the TUG, the effect of facial attractiveness was robust, but the effect of vocal attractiveness was modulated by facial attractiveness. Under the unattractive-face condition, third players with attractive voices increased acceptance rates, while the difference in acceptance between attractive and unattractive voices was not significant under the attractive-face condition. This suggests that the attractiveness effect of faces may have more advantage in fairness decision making compared to the attractiveness effect of voices. This validated our Hypothesis 5 and is in line with previous research [17,18,19]. Thus, multisensory perception of attractiveness exhibits “face dominance”, showing that attractive faces dominate in decision making compared to attractive voices. It is important to note that our study did not directly compare the effects of facial and vocal attractiveness. Future research should address this gap by designing experiments that allow for a direct comparison, helping to determine whether facial attractiveness still holds a distinct advantage in decisions. In addition, in the TDG, where participants were not involved in monetary distribution, the beauty premium of faces was modulated by fairness and vocal attractiveness regarding reasonableness ratings. Unfair offers from proposers who had attractive faces were rated as more reasonable than those who had unattractive faces only when they had unattractive voices. Nonetheless, the effects of vocal attractiveness did not exist. Accordingly, the beauty premium is influenced by different decision-making contexts. Our findings confirm a prior study using two-person games [23], which also confirmed the beauty premium from audiovisual channels and that social interest effects varied across gaming situations.

In addition, no matter whether third players had attractive faces or voices, unfair/fair offers were more likely to be accepted when third players expressed positive social interest than negative social interest in the TUG. The possible reason is that positive social interest releases a signal of “goodwill” that encourages participants to develop approach motivations and engage in more altruistic behaviors [22,31]. So, participants were willing to sacrifice their own interests to accept offers that were unfair to themselves but fair to third players. Whereas unfair allocations from proposers expressing negative social interest (compared to positive social interest) were rated as more reasonable in the TDG. It is noteworthy that there are many differences between the TUG and TDG, such as the game tasks, the holder of face and voice stimuli, the decision processes, and whether participants were interest-free in the game. Hence, it is extremely challenging to compare the TUG and TDG results directly. For this reason, we have generated the following exploratory hypotheses (H7–H9) to be examined in the future:

**H7.**  *The influence of social interest would be different in different decision contexts where other factors are kept the same.*

Additionally, in unfair conditions, participants were more willing to punish proposers expressing positive social interest than negative social interest when proposers’ levels of facial attractiveness and vocal attractiveness mismatched. One potential reason (H8) may be as follows:

**H8.**  *The conflict between facial attractiveness and vocal attractiveness exacerbates participants’ perception of inconsistency between social interest and allocations, leading to harsher punishment to proposers showing positive social interest.*

**H9.**  *Participants’ understandings of “you” may differ. In the TUG, participants had the power for monetary acceptance, thus they may have interpreted the third-party players’ statements as directed at themselves. While in the TDG, monetary payoffs to participants were not involved and participants were just bystanders. They may have assumed that the proposer was talking to the recipient. It is less contradictory for the proposer to say “I don’t like you” to the recipient and then give them an unfair offer. Here, the target of the social interest statement in the TDG is unclear, since we did not test how the participants interpreted the word “you”. Hence, the interpretation of the social interest effects remains speculative and future research should incorporate a test on participants’ interpretation of the term “you” to clearly identify the specific targets of social interest and validate our speculation.*

There are several limitations in the present experiment. Firstly, the voices that convey positive and negative interest are two different sets of voices, not the same voices making different statements, which could result in the cues of social interest being confounded with other features of the voices. Secondly, the convenient manipulation of social interest (the voice saying “I like you” or “I don’t like you”) is blunt and unrealistic. In particular, the target of the social interest statement is unclear and we did not test participants’ understandings of the word “you”. Such ambiguity makes the interpretation of the social interest effects difficult. Future research should adopt more scientific methods to manipulate social interest to clarify the target of social judgment and achieve genuine social interaction. Thirdly, using only the combination of male stimuli and female participants, there were also no measures concerning whether the participants were heterosexual, which may result in limitations to our findings. Future studies could use simpler tasks and expand the gender of materials and subjects, as well as measuring individual sexual orientation, to draw a more comprehensive picture. Fourthly, participants only had multiple pieces of information about one of the people in the game with them, but no information at all about the other person in the game. This left participants to imagine and assume lots of information about that other person; how they filled in those parameters is an uncertainty. Both the proposer’s and the third party’s appearance were important to the fairness judgments. However, since we already had four factors, it was difficult to test more factors. Future research may examine how attractiveness and social interest of all people in the game affect decision making. Furthermore, the present study employed repetitive stimulus in two games. Although we reported the agreements of the outcomes across repetitions for all experimental conditions, this may still produce some carry-over effects.

## 5. Conclusions

The present study demonstrated the effect of facial attractiveness, vocal attractiveness, and social interest on females’ fairness considerations in two three-person bargaining games when participants were involved in money distribution or they acted as an interest-free third party. Although we observed a more generous beauty premium effect, the pro-attractiveness bias was shown to be stronger for faces rather than voices. Moreover, the effects of attractiveness and social interest were heterogenous across different level of fairness and different economic games.

## Figures and Tables

**Table 1:** Means and standard deviations for ratings of vocal attractiveness, pleasure, and arousal (*M* ± *SD*).

Voice	Attractiveness	Pleasure	Arousal
**Attractive voices—positive social interest**	6.37	5.45	5.19
	(0.30)	(1.04)	(0.64)
**Attractive voices—negative social interest**	6.49	5.04	5.01
	(0.29)	(0.68)	(0.62)
**Unattractive voices—positive social interest**	3.45	5.06	4.38
	(0.23)	(0.61)	(0.91)
**Unattractive voices—negative social interest**	3.44	5.35	4.58
	(0.36)	(0.98)	(1.20)

**Table 2:** Means and standard deviations for the proportion of offers accepted in TUG (*M* ± *SD*).

Offer	AAP	AAN	AUP	AUN	UAP	UAN	UUP	UUN
**Fair/Fair**	1.00	0.97	0.99	0.98	0.99	0.96	0.99	0.96
	(0.00)	(0.15)	(0.09)	(0.13)	(0.06)	(0.18)	(0.09)	(0.18)
**Fair/Unfair**	0.94	0.95	0.94	0.96	0.95	0.98	0.95	0.95
	(0.20)	(0.20)	(0.18)	(0.19)	(0.18)	(0.13)	(0.20)	(0.20)
**Unfair/Fair**	0.65	0.26	0.52	0.21	0.51	0.23	0.28	0.22
	(0.44)	(0.38)	(0.45)	(0.36)	(0.45)	(0.36)	(0.40)	(0.39)
**Unfair/Unfair**	0.20	0.18	0.21	0.24	0.19	0.18	0.19	0.15
	(0.37)	(0.36)	(0.39)	(0.37)	(0.35)	(0.34)	(0.37)	(0.31)

**Notes**: AAP = attractive faces, attractive voices, and positive social interest. AAN = attractive faces, attractive voices, and negative social interest. AUP = attractive faces, unattractive voices, and positive social interest. AUN = attractive faces, unattractive voices, and negative social interest. UAP = unattractive faces, attractive voices, and positive social interest. UAN = unattractive faces, attractive voices, and negative social interest. UUP = unattractive faces, unattractive voices, and positive social interest. UUN = unattractive faces, unattractive voices, and negative social interest.

**Table 3:** Means and standard deviations for TDG outcomes (*M* ± *SD*).

Offer	Decision	AAP	AAN	AUP	AUN	UAP	UAN	UUP	UUN
**Fair**	**Reasonableness**	2.24	2.23	2.17	2.13	2.08	2.19	2.08	2.15
		(2.20)	(2.11)	(2.17)	(2.25)	(2.10)	(2.06)	(2.17)	(2.12)
	**Punishment** **Intention**	1.31	1.43	1.40	1.66	1.52	1.81	1.77	1.84
		(1.68)	(1.52)	(1.65)	(1.68)	(1.68)	(1.65)	(1.76)	(1.69)
**Unfair**	**Reasonableness**	−3.22	−1.73	−3.07	−1.74	−3.24	−1.81	−3.27	−1.92
		(1.19)	(2.74)	(1.40)	(2.75)	(1.21)	(2.84)	(1.30)	(2.67)
	**Punishment** **Intention**	4.79	4.41	5.15	4.19	5.47	4.73	5.60	4.99
		(2.60)	(2.88)	(2.57)	(2.97)	(2.62)	(2.88)	(2.62)	(3.03)

**Notes**: Same as in Table 1.

**Table 4:** ANOVA for the proportion of offers accepted in TUG (N = 68).

Factors	*F*	*p*	*η_p_* ^2^
Facial Attractiveness	17.15	<0.001	0.20
Vocal Attractiveness	12.08	0.001	0.15
Social Interest	17.11	<0.001	0.20
Fairness	254.89	<0.001	0.79
Facial Attractiveness × Vocal Attractiveness	5.59	0.021	0.08
Facial Attractiveness × Social Interest	6.61	0.012	0.09
Facial Attractiveness × Fairness	12.64	<0.001	0.16
Vocal Attractiveness × Social Interest	7.41	0.008	0.10
Vocal Attractiveness × Fairness	10.98	<0.001	0.14
Social Interest × Fairness	21.09	<0.001	0.24
Facial Attractiveness × Vocal Attractiveness × Social Interest	0.07	0.787	<0.01
Facial Attractiveness × Vocal Attractiveness × Fairness	0.42	0.685	<0.01
Facial Attractiveness × Social Interest × Fairness	11.45	<0.001	0.15
Vocal Attractiveness × Social Interest × Fairness	5.91	0.003	0.08
Facial Attractiveness × Vocal Attractiveness × Social Interest × Fairness	2.74	0.060	0.04

**Table 5:** ANOVA for reasonableness ratings in TDG (N = 68).

Factors	*F*	*p*	*η_p_* ^2^
Facial Attractiveness	10.01	0.002	0.13
Vocal Attractiveness	0.91	0.345	0.01
Social Interest	10.36	0.002	0.13
Fairness	187.57	<0.001	0.74
Facial Attractiveness × Vocal Attractiveness	0.30	0.587	<0.01
Facial Attractiveness × Social Interest	0.91	0.344	0.01
Facial Attractiveness × Fairness	0.66	0.420	0.01
Vocal Attractiveness × Social Interest	1.45	0.233	0.02
Vocal Attractiveness × Fairness	0.65	0.423	0.01
Social Interest × Fairness	17.20	<0.001	0.20
Facial Attractiveness × Vocal Attractiveness × Social Interest	0.14	0.708	<0.01
Facial Attractiveness × Vocal Attractiveness × Fairness	3.96	0.050	0.06
Facial Attractiveness × Social Interest × Fairness	0.91	0.345	0.01
Vocal Attractiveness × Social Interest × Fairness	0.47	0.494	<0.01
Facial Attractiveness × Vocal Attractiveness × Social Interest × Fairness	0.18	0.674	<0.01

**Table 6:** ANOVA for punishment intention ratings in TDG (N = 68).

Factors	*F*	*p*	*η_p_* ^2^
Facial Attractiveness	39.03	<0.001	0.37
Vocal Attractiveness	16.25	<0.001	0.20
Social Interest	1.21	0.276	0.02
Fairness	188.74	<0.001	0.74
Facial Attractiveness × Vocal Attractiveness	0.43	0.515	<0.01
Facial Attractiveness × Social Interest	0.02	0.889	<0.01
Facial Attractiveness × Fairness	13.19	0.001	0.17
Vocal Attractiveness × Social Interest	2.90	0.093	0.04
Vocal Attractiveness × Fairness	0.08	0.785	<0.01
Social Interest × Fairness	13.95	<0.001	0.17
Facial Attractiveness × Vocal Attractiveness × Social Interest	1.75	0.190	0.03
Facial Attractiveness × Vocal Attractiveness × Fairness	1.07	0.305	0.02
Facial Attractiveness × Social Interest × Fairness	<0.001	0.990	<0.01
Vocal Attractiveness × Social Interest × Fairness	2.45	0.122	0.04
Facial Attractiveness × Vocal Attractiveness × Social Interest × Fairness	13.42	<0.001	0.17

## Data Availability

The data supporting the conclusions of this article are available on the GitLab (https://jihulab.com/attractiveness/beauty-and-social-interest-matter (accessed on 1 December 2014)) or upon reasonable request from the authors.
